# Phosphorylation of E-cadherin at threonine 790 by protein kinase Cδ reduces β-catenin binding and suppresses the function of E-cadherin

**DOI:** 10.18632/oncotarget.9403

**Published:** 2016-05-17

**Authors:** Chien-Lin Chen, Shu-Hui Wang, Po-Chao Chan, Meng-Ru Shen, Hong-Chen Chen

**Affiliations:** ^1^ Department of Life Sciences, National Chung Hsing University, Taichung 402, Taiwan; ^2^ Department of Pharmacology, National Cheng Kung University, Tainan 704, Taiwan; ^3^ Department of Obstetrics and Gynecology, National Cheng Kung University Hospital, Tainan 704, Taiwan; ^4^ Graduate Institute of Biomedical Sciences, National Chung Hsing University, Taichung 402, Taiwan; ^5^ Rong-Hsing Research Center for Translational Medicine, National Chung Hsing University, Taichung 402, Taiwan

**Keywords:** PKCδ, E-cadherin, cell junction, phosphorylation

## Abstract

Proper control of cell-cell adhesion is crucial for embryogenesis and tissue homeostasis. In this study, we show that protein kinase C (PKC)δ, a member of the novel PKC subfamily, localizes at cell-cell contacts of epithelial cells through its C2-like domain in an F-actin-dependent manner. Upon hepatocyte growth factor stimulation, PKCδ is phosphorylated and activated by Src, which then phosphorylates E-cadherin at Thr790. Phosphorylation of E-cadherin at Thr790 diminishes its interaction with β-catenin and impairs the homophilic interaction between the ectodomains of E-cadherin. The suppression of PKCδ by its dominant-negative mutants or specific short-hairpin RNA inhibits the disruption of cell-cell adhesions induced by hepatocyte growth factor. Elevated PKCδ expression in cancer cells is correlated with increased phosphorylation of E-cadherin at Thr790, reduced binding of E-cadherin to β-catenin, and poor homophilic interaction between E-cadherin. Analysis of surgical specimens confirmed that PKCδ is overexpressed in cervical cancer tissues, accompanied by increased phosphorylation of E-cadherin at Thr790. Together, our findings unveil a negative role for PKCδ in cell-cell adhesion through phosphorylation of E-cadherin.

## INTRODUCTION

Adherens junctions are calcium-dependent cell-cell adhesion junctions that are mediated by the transmembrane glycoprotein E-cadherin. The integrity of the complexes between the E-cadherin cytoplasmic domain and catenins has been regarded as a key factor for the homophilic interaction of E-cadherin [1]. The cytoplasmic domain of E-cadherin contains two regions for p120-catenin and β-catenin binding. p120-Catenin binds to the membrane-proximal region of the E-cadherin cytoplasmic domain and promotes the stability of E-cadherin at the cell surface by preventing the endocytosis of E-cadherin [2, 3]. β-catenin binds to the distal region of the E-cadherin cytoplasmic domain and further recruits the actin-binding protein α-catenin, thereby linking adherens junctions to the actin cytoskeleton [4].

E-cadherin binds to β-catenin soon after its synthesis in the endoplasmic reticulum, and the two proteins traffic together to the basolateral membrane [5–7]. *In vitro* phosphorylation of the purified cadherin cytoplasmic domain within a serine cluster region (residues 838-848) by CKII and GSK3β strengthens its affinity for β-catenin [8–11]. Gottardi and colleagues recently narrowed these phosphorylation sites to three residues (S840, S846, and S847) that are required for high-affinity β-catenin binding, cell adhesion, and surface stability of E-cadherin [12]. E-cadherin is phosphorylated at these sites before reaching the cell surface [12], suggesting that cadherin phosphorylation at the serine cluster region may be integral to the E-cadherin-catenin complex formation. Nonetheless, the kinases(s) regulate the phosphorylation at the serine cluster region are not known.

The protein kinase C (PKC) isozymes are serine/threonine protein kinases, which can be classified into classical PKCs (cPKCs), novel PKCs (nPKCs), and atypical PKCs (aPKCs) subfamilies based on their ability to be activated by diacylglycerol and Ca^2+^ [13–15]. PKC isozymes are involved in a wide variety of cell functions, including cell-cell adhesion. For example, the classical PKCα and PKCβ have been reported to regulate the cell-cell junctions and permeability of vascular endothelial cells [16, 17]. Atypical PKC in complex with PAR3 and PAR6 is involved in the regulation of tight junctions [18]. In the nPKCs family, PKCδ is widely expressed in various cell types and tissues and plays a variety of roles in cell proliferation, differentiation, apoptosis and tumor progression [19]. PKCδ has been shown to suppress the function of E-cadherin [20, 21], but the underlying mechanism for this suppression is unclear. In this study, we demonstrate that PKCδ directly phosphorylates E-cadherin at Thr790 upon growth factor stimulation, which decreases the binding of E-cadherin to β-catenin and thereby impairs the homophilic interaction of E-cadherin. Our study provides the first example that the affinity of E-cadherin for β-catenin can be negatively regulated by phosphorylation at a threonine residue that is not located within the serine cluster region of E-cadherin's cytoplasmic domain.

## RESULTS

### PKCδ localizes at cell-cell contacts through its C2-like domain in an F-actin-dependent manner

**Figure 1:**
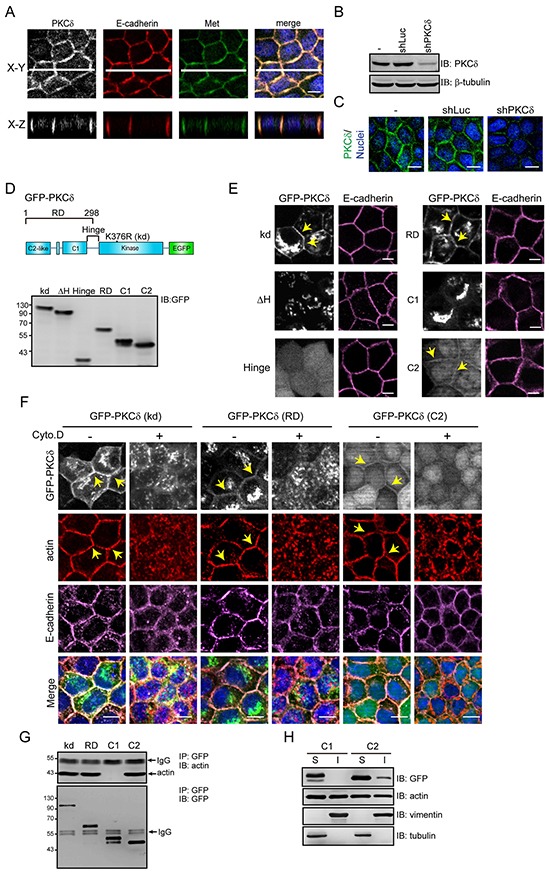
PKCδ localizes at the cell-cell contacts through its C2-like domain in an F-actin-dependent manner **A.** MDCK cells were grown to confluence and were then stained for PKCδ, E-cadherin, Met, and DNA. White lines on the confocal x-y sections represent regions where the confocal x-z sections were taken. The scale bar represents 10 μm. **B.** MDCK cells were infected with recombinant lentiviruses expressing shRNA specific to canine PKCδ (shPKCδ) or to luciferase (shLuc) as a control. The expression levels of PKCδ and β-tubulin (as a loading control) were analyzed by immunoblotting (IB) with the indicated antibodies. **C.** The cells, as in panel (B), were stained for PKCδ and DNA. The scale bar represents 10 μm. **D.** The diagram depicts the domain organization of GFP-PKCδ. The GFP-PKCδ derivatives including the kinase-deficient mutant (kd; K376R), the ΔH mutant with a deletion of the hinge region (a.a. 280-347), the regulatory domain (RD; a.a. 1-298), the C2-like domain (C2; a.a. 1-123), the C1 domain (C1; a.a. 124-298), and the hinge region (a.a. 280-347) were stably expressed in MDCK cells. The expression levels of the GFP-PKCδ derivatives were analyzed by immunoblotting with anti-GFP antibody. **E.** The cells, as in panel (D), were stained with anti-E-cadherin antibody (clone ECCD2). The arrowheads indicate the presence of the GFP-PKCδ kd mutant, the regulatory domain, and the C2-like domain at the cell-cell contacts. The scale bar represents 10 μm. **F.** MDCK cells stably expressing GFP-PKCδ kd mutant, RD, or C2-like domain, were grown to confluence and were then treated with 10 μM cytochalasin D (Cyto. D) for 2 h before they were stained with anti-E-cadherin (clone 36), anti-actin, and DAPI. The arrows indicate the presence of the GFP-PKCδ proteins and F-actin at the cell-cell contacts. The scale bar represents 10 μm. **G.** MDCK cells stably expressing GFP-PKCδ proteins (kd, RD, C1, and C2-like) were grown to confluence before they were lysed. The GFP-PKCδ proteins were immunoprecipiated (IP) by anti-GFP antibody and the immunocomplexes were analyzed by immunoblotting (IB) with antibodies to actin and GFP. **H.** MDCK cells stably expressing GFP-PKCδ C1 domain or C2-like domain were grown to confluence. The cell lysates were fractionated into 1% NP40-soluble (S) and insoluble (I) fractions and analyzed by immunoblotting with the indicated antibodies.

We have previously demonstrated that GFP-fused PKCδ localizes to adherens junctions and the Golgi complexes [20]. However, whether endogenous PKCδ behaves similar to GFP-PKCδ residing at those sites is not clear. To our best knowledge, the localization of endogenous PKCδ has never been described elsewhere. In this study, we demonstrated that endogenous PKCδ was mainly detected at the cell-cell contacts of Madin-Darby canine kidney (MDCK) cells, in which it co-localized with E-cadherin and Met, the hepatocyte growth factor (HGF) receptor (Figure 1A). The depletion of PKCδ by shRNA significantly decreased the fluorescent intensity at the cell-cell contacts (Figure 1B and 1C), which supports the specificity of the fluorescent signals.

To understand the mechanism by which PKCδ localizes to cell-cell contacts, GFP-PKCδ and its mutants were expressed in MDCK cells (Figure 1D). Our results showed that the kinase-deficient (kd) mutant and the regulatory domain of PKCδ strongly resided at the cell-cell contacts and the Golgi complexes (Figure 1E). The regulatory domain of PKCδ consists of the C1 and C2-like domains. The C2-like domain localized to the cell-cell junctions, while the C1 domain localized to the Golgi complexes (Figure 1E). The disruption of the F-actin integrity by cytochalasin prevented the localization of the C2-like domain at the cell-cell contacts (Figure 1F), rendering it possible that the C2-like domain may bind to F-actin at the cell-cell contacts. Indeed, the C2-like domain, but not the C1 domain, was co-precipitated with actin (Figure 1G) and detected in the 1% NP40-insoluble fraction of the cell lysates (Figure 1H). These data together suggest that PKCδ may localize at the cell-cell contacts through its C2-like domain in an F-actin-dependent manner.

### Phosphorylation and activation of PKCδ by Src is essential for HGF to induce cell scattering

**Figure 2:**
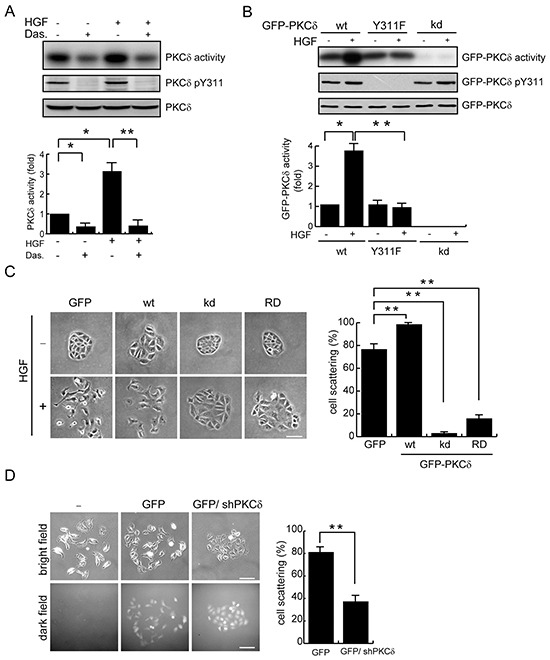
Phosphorylation and activation of PKCδ by Src is important for the scatter of MDCK cells upon HGF stimulation **A.** MDCK cells were serum-starved for 24 h and were then treated with (+) or without (−) the Src inhibitor dasatinib at 100 nM for 1 h before they were stimulated with HGF (20 ng/ml) for 15 min. Endogenous PKCδ was immunoprecipitated using an anti-PKCδ antibody, and the immunocomplexes were analyzed by immunoblotting with antibodies to PKCδ or PKCδ pY311. To measure the PKCδ activity, the immuoncomplexes were subjected to an *in vitro* kinase assay in the presence of [γ-^32^P]ATP and myelin basic protein (MBP) as the substrate. The ^32^P-incorporated MBP were fractionated by SDS-polyacrylamide gel electrophoresis and visualized by autoradiography. The radioisotope activity was quantified using a phosphoimager system. The data are expressed as fold relative to the level of the control. Values (mean ± SD) are from three experiments. *, P < 0.05; **, P < 0.01. **B.** MDCK cells stably expressing GFP-PKCδ wild-type (wt), the Y311F mutant, or the kinase-deficient (kd) mutant were serum-starved for 24 h and were then treated with or without HGF (20 ng/ml) for 15 min. GFP-PKCδ was immunoprecipitated by anti-GFP antibody and the immunocomplexes were subjected to an *in vitro* kinase assay for the PKCδ activity or to immunoblotting with antibodies to GFP and PKCδ pY311. The GFP-PKCδ activity was quantified and expressed as fold relative to the level of the GFP-PKCδ wt. The values (mean ± SD) are from three experiments. *, P < 0.05; **, P < 0.01. **C.** MDCK cells stably expressing GFP-PKCδ or its mutants were allowed to grow as colonies and were then treated with (+) or without (−) HGF (20 ng/ml) for 12 h to induce cell scattering. The percentage of scattered colonies out of the total counted cell colonies (n ≥ 100) was determined. The values (mean ± SD) are from three experiments. **, P < 0.01. Representative micrographs were taken under a phase-contrast microscope. The scale bar represents 50 μm. **D.** MDCK cells were transiently transfected with the pSuperior-GFP or the pSuperior-GFP-siPKCδ plasmid that expresses GFP and shRNA specific to canine PKCδ. The cells were allowed to grow as colonies and were then treated with HGF for 12 h. The percentage of scattered colonies out of the total counted cell colonies expressing GFP (n ≥ 100) was determined. The values (mean ± SD) are from three experiments. **, P < 0.01. Representative micrographs of the cell colonies in both bright and dark fields were taken under an epifluorescence microscope. The scale bar represents 100 μm.

HGF is known for its potent activity to induce disruption of cell-cell adhesions, leading to a “scatter” phenotype of epithelial cells [22]. We found that PKCδ was activated upon HGF stimulation in MDCK cells, accompanied by increased phosphorylation of PKCδ at Tyr311 (Figure 2A). Src can phosphorylate PKCδ at Tyr311 and thereby lead to PKCδ activation under certain circumstances [23, 24]. We showed that the Src inhibitor dasatinib [25] prevented the Tyr311 phosphorylation and activation of PKCδ upon HGF stimulation (Figure 2A). Like endogenous PKCδ, GFP-PKCδ stably expressed in MDCK cells was activated upon HGF stimulation (Figure 2B). In contrast, the Y311F mutant of GFP-PKCδ was unable to be activated by HGF (Figure 2B). The kinase-deficient (kd) mutant was served as a negative control for the *in vitro* kinase assay (Figure 2B). The overexpression of GFP-PKCδ facilitated the HGF-induced cell scattering, whereas the overexpression of the kinase-deficient mutant and the regulatory domain alone, both of which function as dominant-negative mutants, apparently suppressed the cell scattering (Figure 2C). In addition, the depletion of endogenous PKCδ by shRNA inhibited HGF-induced cell scattering (Figure 2D). These results together support the significance of the Met-Src-PKCδ signaling pathway in the disruption of cell-cell contacts upon HGF stimulation.

### PKCδ phosphorylates E-cadherin at Thr790 *in vitro* and in intact cells

**Figure 3:**
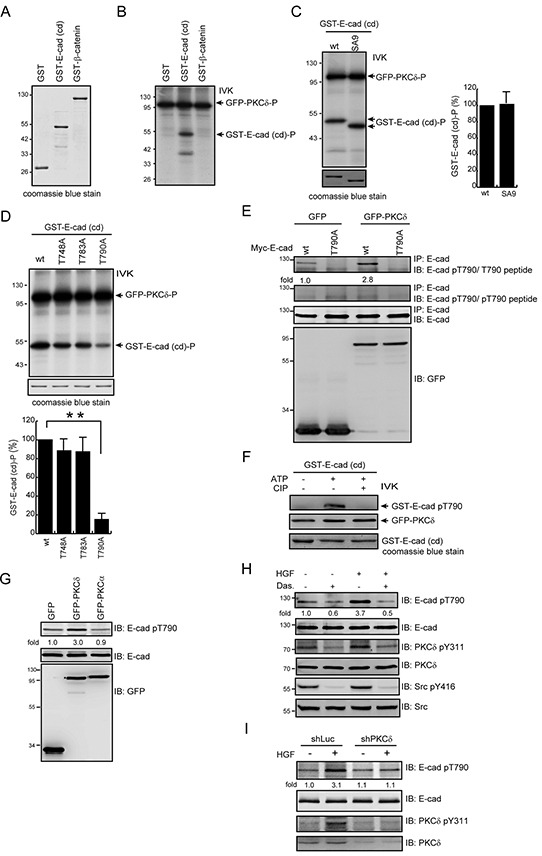
PKCδ phosphorylates E-cadherin at Thr790 in vitro and in intact cells **A.** GST, GST-E-cadherin-cytoplasmic domain (cd), and GST-β-catenin were purified, fractionated by SDS-polyacrylamide gel electrophoresis, and visualized by Coomassie blue staining. **B.** GFP-PKCδ was transiently expressed in HEK293 cells and was then immunoprecipitated using an anti-GFP antibody. The immunocomplexes were subjected to an *in vitro* kinase assay using GST, GST-E-cadherin-cd, or GST-β-catenin as a substrate. The ^32^P-incorporated proteins were fractionated by SDS-polyacrylamide gel electrophoresis and visualized by autoradiography. IVK, *in vitro* kinase assay. **C.** GFP-PKCδ was immunoprecipitated by anti-GFP antibody and the immunocomplexes were subjected to an *in vitro* kinase assay using purified GST-E-cadherin-cd or its SA9 mutant as a substrate. The SA9 mutant consists of nine mutations at the serine resides (S829, S838, S840, S844, S846, S847, S850, S851, and S853) in the highly conserved serine cluster region (a.a. 840-855) of the classical E-cadherins. The radioisotope activity of the ^32^P-incorporated GST-E-cadherin-cd proteins was measured and expressed as the percentage relative to the level of the GST-E-cadherin-cd wt. The values (mean ± SD) are from three experiments. **D.** The GST-E-cadherin-cd or its mutants T748A, T783A, and T790A were purified and served as a substrate for GFP-PKCδ *in vitro*. The ^32^P-incorporated proteins were fractionated by SDS-polyacrylamide gel electrophoresis and visualized by autoradiography. The radioisotope activity of the ^32^P-incorporated GST-E-cadherin-cd proteins was measured and expressed as the percentage relative to the level of the GST-E-cadherin-cd wt. The values (mean ± SD) are from three experiments. **, P < 0.01. **E.** Myc-tagged E-cadherin (Myc-E-cadherin) was transiently co-expressed with GFP or GFP-PKCδ in CHO cells. Myc-E-cadherin was immunoprecipitated by anti-E-cadherin antibody and the immunocomplexes were analyzed by immunoblotting (IB) with anti-E-cad pT790 antibody in the presence of pT790 peptides or T790 peptides. The level of Myc-E-cadherin pT790 was quantified and expressed as fold relative to the level in the cells co-expressing GFP and Myc-E-cadherin wt. **F.** Purified GST-E-cadherin-cd proteins were phosphorylated by GFP-PKCδ in the presence of 1mM ATP and dephosphorylated by CIP in vitro. The phosphorylated and dephosphorylated GST-E-cadherin-cd proteins were analyzed by immunoblotting with anti-E-cad pT790 antibody. **G.** MDCK cells stably expressing GFP, GFP-PKCδ or GFP-PKCα were grown to confluence and then lysed. The cell lysates were analyzed by immunoblotting with antibodies to E-cadherin and E-cadherin pT790. The level of E-cadherin pT790 was quantified and expressed as fold relative to the level in the cells expressing GFP. **H.** MDCK cells were serum-starved for 24 h and were then treated with (+) or without (−) dasatinib (100 nM) and/or HGF (20 ng/ml) for 15 min. To detect E-cadherin pT790, E-cadherin was immunoprecipitated by anti-E-cadherin antibody (clone 36) and the immunocomplexes were analyzed by immunoblotting with antibodies to E-cadherin and E-cadherin pT790. The level of E-cadherin pT790 was quantified and expressed as fold relative to the level of the control. **I.** MDCK cells stably expressing shRNA specific to canine PKCδ were serum-starved for 24 h and then treated with or without HGF (20 ng/ml) for 15 min. The cell lysates were analyzed by immunoblotting with the indicated antibodies. The level of E-cadherin pT790 was quantified and expressed as fold relative to the level of the cells expressing shPKCδ without HGF treatment.

We hypothesized that upon activation at the cell-cell junctions, PKCδ may directly phosphorylate E-cadherin and/or other junctional proteins, leading to disruption of the cell-cell junctions. We demonstrated *in vitro* that PKCδ directly phosphorylated the cytoplasmic domain of E-cadherin but not β-catenin (Figure 3A and 3B). To identify the phosphorylation site for PKCδ in E-cadherin, all serine residues (S829, S838, S840, S844, S846, S847, S850, S851, and S853) in the highly conserved serine cluster region of the classical E-cadherins were substituted with alanine. However, the substitution did not decrease *in vitro* phosphorylation of the cytoplasmic domain of E-cadherin by PKCδ (Figure 3C). Our *in silico* analysis (http://scansite3.mit.edu) revealed that Thr790 is the only potential phosphorylation site for PKCδ. Indeed, the substitution of Thr790 with Ala significantly (~80%) decreased *in vitro* phosphorylation of E-cadherin cytoplasmic domain by PKCδ (Figure 3D).

To facilitate the detection of Thr790-phosphorylated E-cadherin, a phospho-specific antibody (anti-E-cad pT790) was generated. The transient co-expression of Myc epitope-tagged E-cadherin (Myc-E-cadherin) and GFP-PKCδ in CHO cells led to an increase in Thr790 phosphorylation of Myc-E-cadherin but not its T790A mutant (Figure 3E). The specificity of this anti-E-cad pT790 antibody was verified by phospho-T790 peptide (Figure 3E) and GST-E-cadherin cytoplasmic domain that was phosphorylated by PKCδ and dephosphorylated by calf intestine alkaline phosphatase (CIP) *in vitro* (Figure 3F). The stable overexpression of GFP-PKCδ but not GFP-PKCα increased the Thr790 phosphorylation of endogenous E-cadherin in MDCK cells (Figure 3G). In addition, Gö6976, a selective inhibitor for the classical PKC isozymes, did not inhibit the phosphorylation of E-cadherin Thr790 and the scatter of MDCK cells upon HGF stimulation (data not shown). In contrast, the increased phosphorylation of E-cadherin Thr790 was inhibited by the Src inhibitor dasatinib (Figure 3H) and PKCδ depletion (Figure 3I). These data together indicate that upon HGF stimulation, Src-mediated activation of PKCδ leads to an increase in the phosphorylation of E-cadherin at Thr790 in intact cells.

### Phosphorylation of E-cadherin at Thr790 impairs the homophilic interaction of E-cadherin

**Figure 4:**
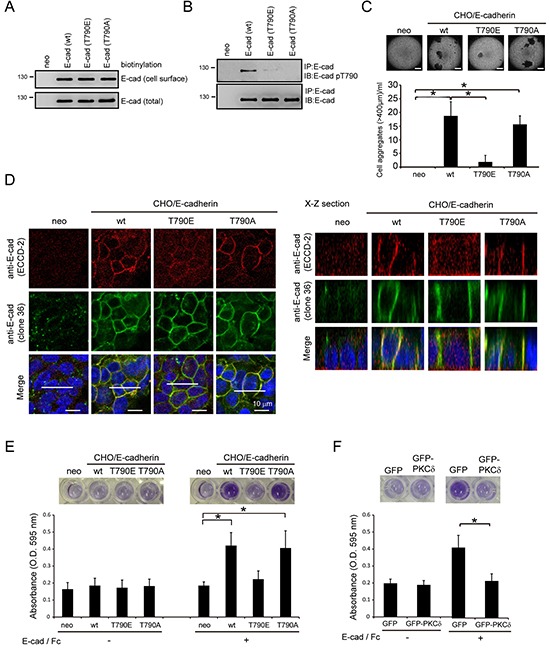
Phosphorylation of E-cadherin at Thr-790 by PKCδ impairs the homophilic interaction of E-cadherin **A.** CHO cells stably expressing E-cadherin or its mutants (T790E and T790A) and their neomycin-resistant control cells (neo) were established by a lentiviral expression system. Those cells were subjected to biotinylation with sulfo-NHS-biotin. For measurement of the cell surface level of E-cadherin, equal amounts of cell lysates were incubated with avidin-immobilized agarose beads and then the complexes were analyzed by immunoblotting with anti-E-cadherin. For measurement of the total expression level of E-cadherin, equal amounts of cell lysates were analyzed by immunoblotting with anti-E-cadherin (clone 36). **B.** CHO cells, as in panel (A), were grown to confluence and then lysed. E-cadherin was immunoprecipitated by anti-E-cadherin (clone 36) and the immunocomplexes were analyzed by immunoblotting with antibodies to E-cadherin and E-cadherin pT790. **C.** CHO cells, as in panel (A), were collected by trypsinization, suspended in medium with 10% serum, and subjected to a constant rotation at 0.5 xg. Two days later, the number of cell aggregates 400 μm or larger in diameter was measured under a phase-contrast microscope. The values (mean ± SD) are from three experiments. *, P < 0.05. Representative micrographs of the cell aggregates were taken under a phase-contrast microscope. The scale bar represents 400 μm. **D.** CHO cells, as in panel (A), were grown to confluence and were then stained with anti-E-cadherin antibodies (clone 36 and ECCD-2). The scale bar represents 10 μm. The X-Z sections along the white lines were shown on the left. **E.** CHO cells (5×10^5^), as in panel (A), were suspended in serum-free medium and then plated onto a 96-well plate coated with or without purified E-cadherin/Fc chimera proteins composed of the extracellular domain of human E-cadherin (amino acids 1-707) fused to the Fc region of human IgG1. Two hours later, the cells were stained with MTT and the absorbance at 595 nm was measured. The values (mean ± SD) are from three experiments. *, P < 0.05. **F.** The adhesion of MDCK cells stably expressing GFP or GFP-PKCδ to purified E-cadherin/Fc chimera protein was analyzed as described in panel (E). The values (mean ± SD) are from three experiments. *, P < 0.05.

Chinese hamster ovary (CHO) cells that do not express E-cadherin were used as a platform to study the function of human E-cadherin (Figure 4). The ectopic expression levels of human E-cadherin and its mutants were comparable in the whole cell lysates and on the cell surface of CHO cells (Figure 4A). The ectopically expressed human E-cadherin, but not T790A or T790E mutants, was detected to be phosphorylated at Thr790 (Figure 4B). The expression of human E-cadherin, but not the phospho-mimetic T790E mutant, allowed CHO cells to form cell aggregates in suspension (Figure 4C). The extent of the cell aggregation induced by the T790A mutant was similar to that induced by the wild-type (wt) E-cadherin (Figure 4C). The monoclonal antibody (clone 36) that recognizes the cytoplasmic domain of E-cadherin was used to detect the subcellular localization of E-cadherin. We found that E-cadherin and T790 mutants were detected at the cell-cell contacts by the clone 36 antibody (Figure 4D), suggesting that the localization of E-cadherin to the cell-cell contacts may not be affected by Thr790 phosphorylation. The monoclonal antibody ECCD-2 recognizes the extracellular domain of E-cadherin only when E-cadherin forms homophilic interactions [20]. Notably, the ECCD-2 antibody detected E-cadherin and the T790A mutant, but not the T790E mutant, at the cell-cell contacts (Figure 4D), suggesting that the phosphorylation of E-cadherin at Thr790 may impair the homophilic interaction of E-cadherin at cell-cell contacts. To further examine this possibility, the binding of the E-cadherin-expressed CHO cells to purified E-cadherin/Fc chimera proteins composed of the extracellular domain of human E-cadherin (amino acids 1-707) fused to the Fc region of human IgG1 was analyzed. Our results showed that the CHO cells expressing the T790E mutant were less effective at binding to the E-cadherin/Fc chimera protein than the cells expressing wt E-cadherin or the T790A mutant (Figure 4E). Moreover, the stable expression of GFP-PKCδ in MDCK cells significantly decreased their binding to the E-cadherin/Fc chimera proteins (Figure 4F). These data together support our notion that phosphorylation of E-cadherin Thr790 by PKCδ impairs the homophilic interaction of E-cadherin.

### Phosphorylation of E-cadherin at Thr790 diminishes its interaction with β-catenin

**Figure 5:**
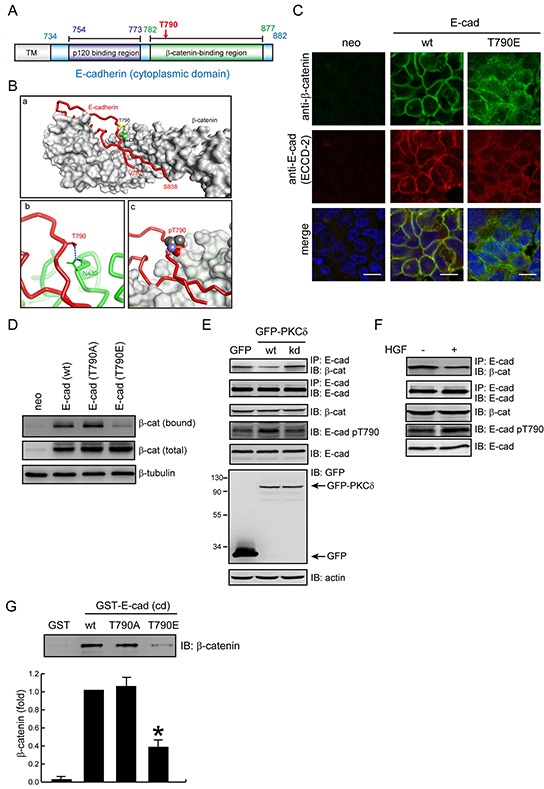
Phosphorylation of E-cadherin at Thr-790 diminishes its interaction with β-catenin **A.** The diagram depicts the regions of E-cadherin for p120-catenin and β-catenin binding. Note that Thr790 resides in the β-catenin binding region. **B.** Simulation for the interface between E-cadherin and β-catenin. (a) The structure of the E-cadherin/β-catenin complex [Protein Data Bank (PDB) ID code: 1I7X]. E-cadherin (a.a 782-838) is represented with a red tube and β-catenin is shown as a surface representation (light grey). The T790 residue of E-cadherin is represented by a yellow stick and β-catenin N430 is represented by green space filling. (b) A close-up view of the interaction of β-catenin N430 (represented by a green stick) with E-cadherin T790 (represented by a red stick). Hydrogen bonds are shown as blue dashed lines. (c) The side chain of pT790 (shown in space filling representation) clashes with β-catenin (shown in surface representation). **C.** CHO cells stably expressing E-cadherin wt or T790E were grown to confluence and were then stained with anti-β-catenin and anti-E-cadherin (ECCD-2). Note that β-catenin is less organized at the cell-cell contacts of the CHO cells expressing E-cadherin T790E. **D.** CHO cells, as in Figure 4A, were grown to confluence and then were subjected to cell surface biotinylation. For measurement of the membrane bound level of β-catenin, equal amounts of cell lysates were incubated with avidin-immobilized agarose beads and the complexes were analyzed by immunoblotting with anti-β-catenin. For measurement of the total expression level of β-catenin, equal amounts of cell lysates were analyzed by immunoblotting with anti-β-catenin. **E.** MDCK cells stably expressing GFP, GFP-PKC (wt) or (kd) mutant were grown to confluence and then lysed. Equal amounts of cell lysates were immunoprecipitated by anti-E-cadherin antibody (clone 36) and the immunocomplexes were analyzed by immunoblotting with antibodies to β-catenin and E-cadherin. The whole cell lysates were analyzed by immunoblotting with the indicated antibodies. **F.** MDCK cells were treated with (+) or without (−) HGF (20 ng/ml) for 1 hr. Equal amounts of cell lysates were immunoprecipitated by anti-E-cadherin antibody (clone 36) and the immunocomplexes were analyzed by immunoblotting with antibodies to β-catenin and E-cadherin. The whole cell lysates were analyzed by immunoblotting with the indicated antibodies. **G.** Purified β-catenin was incubated with purified GST-E-cadherin-cytoplasmic domain (cd) or GST as a control. The protein complexes were pulled-down by glutathione agarose beads. After washing, the protein complexes were analyzed by immunoblotting with anti-β-catenin. The level of bound β-catenin was quantified and expressed as the fold relative to the level in the GST-E-cadherin-cd wt. The values (mean ± SD) are from three experiments. *, P < 0.05.

The Thr790 residue resides within the region I (residues 782-792) of E-cadherin cytoplasmic domain for β-catenin binding (Figure 5A). The crystal structure of the β-catenin-E-cadherin complex [9] reveals that hydrogen bonding is formed between the Thr790 of E-cadherin and the Asn430 of β-catenin (Figure 5B). This interaction will no longer exist if Thr790 is phosphorylated, because the phosphate group is too large to be accommodated in the interface, and would electrostatically repel the Asn430 of β-catenin (Figure 5B). Indeed, we found that β-catenin was less organized at the cell-cell junctions of the CHO cells expressing the E-cadherin T790E mutant compared to those expressing the wt E-cadherin (Figure 5C and Supplementary Figure S2). In addition, the E-cadherin T790E mutant bound much less to β-catenin than wt E-cadherin did on the cell surface of CHO cells (Figure 5D). In MDCK cells, the phosphorylation of E-cadherin at Thr790 was increased by PKCδ overexpression (Figure 5E) or HGF stimulation (Figure 5F), which was correlated with a decreased interaction of E-cadherin with β-catenin. Moreover, the *in vitro* binding between purified β-catenin and E-cadherin was decreased when the Thr790 of E-cadherin was substituted with Glu (Figure 5G). These results together suggest that the phosphorylation of E-cadherin at Thr790 may impair its interaction with β-catenin.

### Increased expression of PKCδ suppresses the function of E-cadherin in cervical cancers

**Figure 6:**
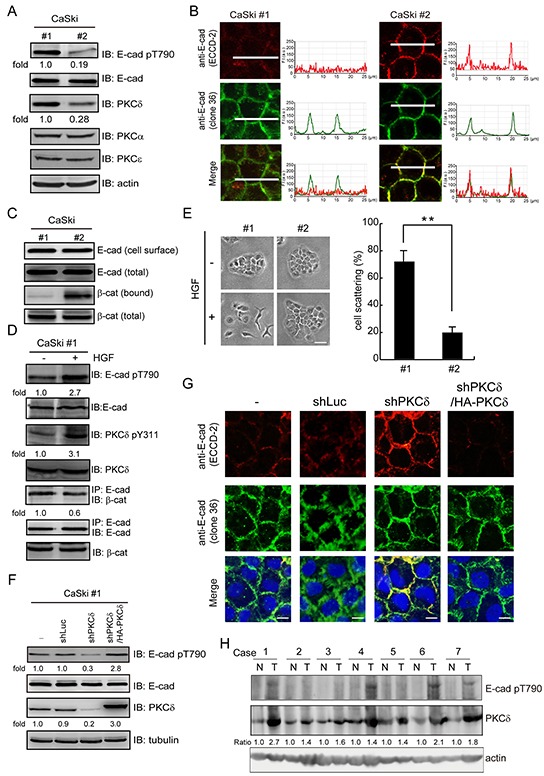
The expression of PKCδ is correlated with Thr790 phosphorylation of E-cadherin in cervical carcinoma **A.** Two subclones (#1 and #2) of human cervical carcinoma CaSki cells were selected on the basis of the PKCδ expression level. The cell lysates were analyzed by immunoblotting with the indicated antibodies. The level of E-cadherin pT790 and PKCδ were quantified and expressed as fold relative to the level in the CaSki #1 cells. **B.** The cells, as in panel (A), were grown to confluence and were then stained with anti-E-cadherin antibodies (clone 36 and ECCD-2). The profiles of the E-cadherin fluorescence intensity (F. I.; a.u. arbitrary units) along the white lines were shown with line graphs. The scale bar represents 10 μm. **C.** Two subclones (#1 and #2) of CaSki cells were grown to confluence and then subjected to cell surface biotinylation. For measurement of the membrane bound level of β-catenin, equal amounts of cell lysates were incubated with avidin-immobilized agarose beads and the complexes were analyzed by immunoblotting with anti-β-catenin and anti-E-cadherin. For measurement of the total expression level of E-cadherin and β-catenin, equal amounts of cell lysates were analyzed by immunoblotting with anti-E-cadherin and anti-β-catenin. **D.** CaSki #1 cells were treated with (+) or without (−) HGF (30 ng/ml) for 15 min. Equal amounts of cell lysates were immunoprecipitated by anti-E-cadherin antibody (clone 36) and the immunocomplexes were analyzed by immunoblotting with antibodies to β-catenin and E-cadherin. The level of E-cadherin pT790, PKCδ pY311 and the β-catenin were measured and expressed as the fold relative to the level of the control cells. **E.** Two subclones (#1 and #2) of CaSki cells were allowed to grow as colonies and treated with or without HGF for 12 h. The percentage of scattered colonies out of the total counted cell colonies (n ≥ 100) was determined. The values (mean ± SD) are from three experiments. **, P < 0.01. **F.** CaSki #1 cells were infected with lentiviruses expressing shRNAs to PKCδ (shPKCδ) or luciferase (shLuc) as a control. HA epitope tagged-PKCδ (HA-PKCδ) was re-expressed in the cells whose endogenous PKCδ had been depleted. The cell lysates were analyzed by immunoblotting with the indicated antibodies. The level of E-cadherin pT790 was measured and expressed as fold relative to the level of the control cells. **G.** The cells, as in panel (F), were grown to confluence and were then stained with anti-E-cadherin antibodies (clone 36 and ECCD-2). The scale bar represents 10 μm. **H.** Expression of PKCδ and phosphorylation of E-cadherin at Thr790 in early-stage cervical cancer. Cervical cancer with pair-frozen tissues of carcinoma and adjacent noncancer epithelia were analyzed by immunoblotting. N, noncancer epithelia; T, tumor tissues. Expression of PKCδ was normalized against actin.

Two subclones (#1 and #2) of human cervical carcinoma CaSki cells were selected on the basis of the PKCδ expression level (Figure 6A). We found that the CaSki clone #1 cells with higher PKCδ expression displayed higher Thr790 phosphorylation of E-cadherin but less homophilic interaction of E-cadherin and its interaction with β-catenin (Figure 6A-6C). Upon HGF stimulation, the PKCδ Tyr311 phosphorylation and the E-cadherin Thr790 phosphorylation in the CaSki clone #1 cells were increased, but the interaction between E-cadherin and β-catenin was decreased (Figure 6D). The clone #1 cells exhibited a more scattered phenotype than the clone #2 cells in response to HGF stimulation (Figure 6E). The depletion of PKCδ in the CaSki clone #1 cells reduced the Thr790 phosphorylation of E-cadherin (Figure 6F) and enhanced the homophilic interactions of E-cadherin (Figure 6G and Supplementary Figure S3). The effects of PKCδ depletion on the Thr790 phosphorylation and the homophilic interaction of E-cadherin were reversed by the re-expression of HA-tagged PKCδ (Figure 6F and 6G). To examine the clinical relevance, the expression of PKCδ and the phosphorylation of E-cadherin at Thr790 were analyzed in the surgical specimens of cervical cancer (Figure 6H). Compared with that of noncancerous tissues, the expression of PKCδ was increased in tumor tissues in all cases. E-cadherin Thr790 phosphorylation was detected in 57% of tumor tissues. These results together support that increased expression of PKCδ in cancer cells may suppress the function of E-cadhern through its phosphorylation at E-cadherin Thr790.

## DISCUSSION

Cell-cell adhesion is modulated in response to extracellular cues. Src has been shown to play an important role in the regulation of cell-cell adhesions [26, 27]. Several junctional proteins are known to be the substrates for Src and their phosphorylation by Src usually has an adverse impact on cell-cell adhesion. For example, the phosphorylation of β-catenin Tyr654 by Src reduces the interaction of β-catenin and E-cadherin [28]. In addition, the phosphorylation of E-cadherin Tyr753 and Tyr754 by Src recruits the binding of Hakai, a Cbl-like E3 ubiquitin ligase, leading to ubiquitination and endocytosis of the E-cadherin-β-catenin complex [29]. In this study, we discover a novel route for Src to suppress cell-cell adhesion through PKCδ. Upon activation by Src, PKCδ phosphorylates E-cadherin at Thr790, which diminishes its binding to β-catenin and leads to suppression of the homophilic interaction of E-cadherin. The significance of this Src-PKCδ-E-cadherin axis in cell-cell adhesion is demonstrated by dominant-negative PKCδ mutants and PKCδ depletion (Figure 2C and 2D), both of which inhibit the disruption of cell-cell adhesion triggered by HGF. The C2-like domain of PKCδ has been proposed to bind to F-actin in neutrophils [30]. In this study, we found that PKCδ localizes at the cell-cell contacts of MDCK cells through its C2-like domain in an F-actin-dependent manner (Figure 1).

In this study, an antibody specifically recognizing Thr790-phosphorylated E-cadherin was generated (Figure 3E and 3F). Using this antibody, we show that phosphorylation of E-cadherin at Thr790 is increased upon overexpression of PKCδ but not PKCα (Figure 3G). In addition, phosphorylation of E-cadherin at Thr790 is increased in response to HGF stimulation (Figure 3H), which is suppressed by depletion of PKCδ (Figure 3I). Evidence that E-cadherin Thr790 is phosphorylated in intact cells will be strengthened by mass spectrometry, which is currently underway. Nonetheless, negative charge substitution at this site diminishes cell-cell adhesion (Figure 4) and β-catenin binding (Figure 5), supporting that E-cadherin phosphorylation at Thr790 has an adverse impact to β-catenin binding.

The cadherin cytoplasmic domain is not structured in the absence of β-catenin [31], and binds in an extended conformation that forms a large interface with β-catenin [9]. This mode of binding may allow for local alterations of the interaction without affecting the rest of the interface. In this manner, posttranslational modifications like phosphorylation can modulate the interaction in a graded fashion rather than serving as a simple on/off switch. Based on the crystal structure of the β-catenin-E-cadherin complex, the cytoplasmic domain of E-cadherin can be divided into five regions for β-catenin binding [9]. Region I (residues 782-792) starts as an extended polypeptide within the β-catenin groove at arm repeats 7-9, running parallel to the H3 helices and toward the β-catenin COOH-terminus. E-cadherin Thr790 forms a hydrogen bond with β-catenin Asn430 (Figure 5B). Phosphorylation of Thr790 likely causes the region I to dissociate from β-catenin because the phosphate group is too large to be accommodated in the interface, and would electrostatically repel the Asn430 of β-catenin (Figure 5B). Similarly, E-cadherin Asp819 in the region II (resides 793-820) forms a hydrogen bond with β-catenin Tyr654 [9]. Phosphorylation of β-catenin Tyr654 by Src causes a 6-fold reduction in the affinity of β-catenin for E-cadherin [28] likely because of the dissociation of the region II from β-catenin.

The region IV (residues 838-848) of E-cadherin's cytoplasmic domain contains eight serine residues in consensus positions for CKII- and GSK3β-mediated phosphorylation. In vitro phosphorylation of the purified cadherin tail by CKII and GSK3β strengthens its affinity for β-catenin ~800-fold by creating an additional interaction surface [8–11]. Gottardi and colleagues recently narrowed these phosphorylation sites to three residues (S840, S846, and S847) that are required for high-affinity β-catenin binding, cell adhesion, inhibition of cell migration, and surface stability of E-cadherin in cultured cells [12]. E-cadherin is phosphorylated at these sites before reaching the cell surface [12], suggesting that cadherin phosphorylation at the region IV may be integral to the E-cadherin-catenin complex formation, surface stability, and cell-cell adhesion. In this study, we show that endogenous PKCδ mainly localizes at cell-cell junctions through its C2-like domain in an F-actin-dependent manner (Figure 1). Upon its activation, PKCδ directly phosphorylates E-cadherin at Thr790, leading to a decrease in the interaction of E-cadherin with β-catenin. Our study provides the first example that the affinity of E-cadherin for β-catenin can be negatively regulated by phosphorylation at a threonine residue in the region I of E-cadherin's cytoplasmic domain in response to extracellular cues.

p120-Catenin binds a membrane-proximal region of E-cadherin tail and is generally viewed as the master regulator of cadherin surface stability by occluding an acidic residue-rich endocytosis signal [3, 32]. β-Catenin binds the E-cadherin tail more distally and has been proposed to be critical for cadherin surface stability. Deletion of the β-catenin-binding domain or mutation of S840, S846, and S847 to Ala in the serine cluster region leads to E-cadherin accumulation within the trans-Golgi network, early endosomes, and lysosomes [12, 33]. β-Catenin binding may render the conserved ubiquitination and proteasomal degradation motif L-S^846^-S^847^-L within the PEST sequence of E-cadherin's cytoplasmic tail inaccessible [9, 31]. However, modifications that affect E-cadherin/β-catenin binding more modestly and/or that do not involve the serine cluster region, such as phosphorylation of β-catenin at Tyr654 [28], clearly affect E-cadherin function without obviously affecting cadherin surface levels [34, 35]. Similarly, we found that phosphorylation of E-cadherin at Thr790 does not affect cadherin surface levels (Figure 4).

A special set of monoclonal antibodies was generated to distinguish the inactive and active states of E-cadherin at the cell surface [36]. The monoclonal anti-E-cadherin antibody ECCD-2 used in this study preferentially recognizes “active” E-cadherin when homophilic interactions between their ectodomains are formed [20]. How does the phosphorylation of E-cadherin at Thr790 impair the homophilic interaction between the extracellular domains of E-cadherin? β-Catenin binds E-cadherin and recruits the F-actin-binding protein α-catenin, coordinating cadherins with the cortical actin cytoskeleton [37]. Previous studies show that the binding of catenins to the cytoplasmic domain of cadherin regulates the dimerization state and/or clustering of cadherin, as well as the ability of cadherin to form homophilic interaction [38–41]. Therefore, the possibility exists that the phosphorylation of E-cadherin at Thr790 by PKCδ decreases the affinity of E-cadherin for β-catenin, which may affect the conformation of the extracellular domain through an inside-out cytoskeleton-dependent mechanism and thereby impair the homophilic interaction of E-cadherin.

Suppression of the expression and/or function of E-cadherin is a hallmark of epithelial-to-mesenchymal transition during carcinogenesis. We found in this study that an increased level of PKCδ in cervical carcinoma CaSki cells is correlated with an increase in the phosphorylation of E-cadherin Thr790 and poor homophilic interaction of E-cadherin. PKCδ is often found to be overexpressed in malignant tumors [42–46]. In this study, our analysis of surgical specimens confirmed that PKCδ was overexpressed in cervical cancer tissues, accompanied by increased phosphorylation of E-cadherin at Thr790. In summary, this work not only unveils a novel role for PKCδ in cell-cell adhesion but also highlights the significance of PKCδ in malignant tumor progression.

## MATERIALS AND METHODS

### Materials

The rabbit polyclonal antibody specific to E-cadherin pT790 was generated using synthesized phospho-peptides C-RNDVAPCpTLMS (pT790 peptide) as the antigen (GeneTex, Inc., Hsinchu, Taiwan). The control C-RNDVAPCTLMS peptides (T790 peptide) were provided by GeneTex, Inc. The rabbit polyclonal anti-PKCδ antibodies (C-17 for immunofluorescence staining and C-20 for immunoblotting) and the mouse monoclonal anti-tubulin and anti-GFP (B-2) antibodies were purchased from Santa Cruz Biotechnology (Santa Cruz, CA). The mouse monoclonal anti-GFP (clone 9) antibody for immunoprecipitation was purchased from Roche. The mouse monoclonal anti-E-cadherin (clone 36 for immunofluorescent staining and clone 34 for immunoblotting), and anti-PKCδ (clone 14 for endogenous PKCδ in CHO cells) antibodies and the rabbit polyclonal anti-PKCα antibody were purchased from BD Transduction Laboratories (Franklin Lakes, NJ). The rabbit polyclonal anti-PKCδ pY311 and anti-Src pY416 antibodies were purchased from Cell Signaling Technology (Danvers, MA). The rat monoclonal anti-E-cadherin (clone ECCD-2) antibody was purchased from Invitrogen Life Technologies. The mouse monoclonal anti-Met (DL-21) antibody was purchased from Upstate Biotechnology (Lake Placid, NY). The E-cadherin/Fc chimera human recombinant protein was purchased from R&D Systems (Minneapolis, MN). The recombinant human HGF was purchased from PeproTech (Rocky Hill, NJ). Protein-A-Sepharose and Glutathione-Sepharose were purchased from GE Healthcare Life Sciences. EZ-Link sulfo-NHS-biotin and avidin-immobilized agarose beads were purchased from Pierce (Rockford, IL, USA). Src inhibitor Dasatinib was purchased from BioVision (Milpitas, USA). Purified β-catenin was purchased from OriGene Technologies (Rockville, MD, USA). Calf intestinal alkaline phosphatase was purchased from New England Biolabs.

### Plasmids and mutagenesis

The pEGFP-N1-PKCα plasmid was kindly provided by D. Joubert and has been previously described [47].

The plasmid pHACE-PKCδ (HA-PKCδ) was kindly provided by Dr. Jae-Won Soh and has been described previously [48]. The pEGFP-N1-PKCδ plasmid and the pEGFP-N2-PKCδ plasmids encoding the kinase-deficient mutant (kd), the regulatory domain (RD), and the mutant with a deletion at the hinge region (ΔH) have been described previously [20]. To construct pEGFP-N2-PKCδ encoding the C2-like domain (a.a. 1-159), the C1 domain (a.a. 124-298), and the hinge region (a.a. 280-347), the corresponding cDNA fragments were amplified by polymerase chain reaction (PCR) with specific primers using pEGFP-N1-PKCδ as the template and ligated in-frame to the pEGFP-N2 vector *via* the SmaI site. For the C2-like domain, the forward primer 5′-ATCATGGCACCCTTCCTG-3′ and the reverse primer 5′-GTTCTTGATCTAGTGGATCTTGGCCTG-3′ were used. For the C1 domain, the forward primer 5′-ATGGATGGGGATTGCAAA-3′ and the reverse primer 5′-GGAGAATTCAGATCTCTGGGTCACTTG-3′ were used. For the hinge region, the forward primer 5′-GGTATCAACCAAAAGCTCTTGGCTGAG-3′ and the reverseprimer 5′-GAAGTTCTCAAGGGTGCACCG-3′ were used.

The pcDNA6/His-E-cadherin plasmid was kindly provided by Dr. Mien-Chie Hung (University of Texas M.D. Anderson Cancer Center, Houston, TX) and has been described previously [49]. To construct E-cadherin into the lentiviral vector pLKO.AS2.neomycin (National RNAi Core Facility, Academia Sinica, Taiwan), the corresponding cDNA fragment of the full-length E-cadherin was amplified by PCR with the forward primer 5′-AAAGCTAGCATGGGCCCTTGGAGCCGCAGCCTC-3′, and the reverse primer 5′-CGTTTAAACCTAGTCGTCCTCGCCGCCTCCGTA-3′ (restriction enzyme sites are underlined) using pcDNA6/His-E-cadherin as the template and ligated in-frame to the pLKO.AS2.neomycin vector by the NheI and PmeI sites. For the expression of the glutathione-*S*-transferase (GST)-fused cytoplasmic domain (a.a. 734-882) of E-cadherin, the corresponding cDNA fragment of the E-cadherin cytoplasmic domain was amplified by PCR with the forward primer 5′-GGCGGATCCATGAGAGCGGTGGTCAAAGAGCCC-3′ and the reverse primer 5′-GGCGAATTCTCGTCCTCGCCGCCTCCGTACATG-3′ (restriction enzyme sites are underlined) using pcDNA6/His-E-cadherin as the template and ligated in-frame to the pGEX-2T vector *via* the BamHI and EcoRI sites. The PKCδ Y311F mutant and the E-cadherin mutants including T790A, T790E, and SA9 (S829A, S838A, S840A, S844A, S846A, S847A, S850A, S851A, and S853A) mutants were generated using a QuikChange site-directed mutagenesis kit (Stratagene). The desired mutations were confirmed by dideoxy DNA sequencing, a service provided by the Biotechnology Center of the National Chung Hsing University, Taiwan.

### Cell culture and transfection

For transient transfection, the cells were seeded on 60-mm culture dishes for 18 h and were then transfected with plasmids using Lipofectamine. To generate MDCK cells stably expressing GFP-PKCδ or its mutants, MDCK cells were transfected with GFP-PKCδ or mutants by Lipofectamine. Two days after transfection, the cells were selected in medium containing 0.5 mg/ml neomycin G418. Ten days later, the neomycin resistant cells were pooled and analyzed for exogenous PKCδ expression by immunoblotting using anti-GFP. To generate CHO cells stably expressing E-cadherin or its mutants, CHO cells were infected with lentiviruses encoding E-cadherin or its mutants for 24 h and were subsequently selected in the growth medium containing 0.5 mg/ml G418. To isolate subclones (#1 and #2) of CaSki cells, 50 cells were seeded in 100-mm culture dish and allowed to grow as colonies. Ten days later, the cell colonies were picked up by a cylinder and were selected on the basis of the PKCδ expression level.

### Short-hairpin RNA (shRNA) and lentiviral production

The pSuperior-GFP-siPKCδ expressing GFP and shRNA to canine PKCδ has been described previously [50]. The lentiviral expression system and the pLKO-AS1-puromycin (puro) plasmid encoding shRNAs were obtained from the National RNAi Core Facility (Academia Sinica, Taiwan). The target sequences for canine PKCδ and human PKCδ were 5′-CAAGGCTACAAATGCAGGCAA-3′ and 5′-GGCCGCTTTGAACTCTACCGT-3′, respectively. To produce lentiviruses, HEK293T cells were cotransfected with 2.25 μ g pCMV-ΔR8.91, 0.25 μg pMD.G, and 2.5 μg pLKO-AS1-puro-shRNA (or pLKO-AS2-neo-E-cadherin) using Lipofectamine. After 3 days, the medium containing lentivirus particles was collected and stored at −80°C. The cells were infected with the lentiviruses encoding shRNAs for 24 h and were subsequently selected in the growth medium containing 2.5 μg/ml puromycin. The cells infected with the lentiviruses encoding E-cadherin were selected in the growth medium containing 0.5 mg/ml G418.

### Immunoprecipitation and immunoblotting

Cells were lysed in 1% Nonidet P-40 lysis buffer (1% Nonidet P-40, 20 mM Tris-HCl, pH 8.0, 137 mM NaCl, 10% glycerol and 1 mM Na_3_VO_4_) containing protease inhibitors (1 mM phenylmethylsulfonyl fluoride, 0.2 trypsin inhibitory units/ml aprotinin, and 20 μg/ml leupeptin). For immunoprecipitation, aliquots of cell lysates were incubated with primary antibodies for 1.5 hours at 4°C. Immunocomplexes were collected on protein-A Sepharose beads. For monoclonal antibodies, protein-A Sepharose beads were coupled with rabbit anti-mouse IgG (1 μg) before use. The beads were washed three times with 1% Nonidet P-40 lysis buffer, boiled for 3 minutes in SDS sample buffer, subjected to SDS-polyacrylamide gel electrophoresis, and transferred to nitrocellulose (Schleicher and Schuell). Immunoblotting was performed with appropriate antibodies using the Amersham Biosciences enhanced chemiluminescence system for detection. Chemiluminescent signals were detected and quantified by the Fuji LAS-3000 luminescence image system.

### *In vitro* kinase assay

To perform the in vitro kinase assays for PKCδ or GFP-PKCδ, the immunoprecipitates by anti-PKCδ or anti-GFP were washed three times with 1% Nonidet-40 lysis buffer and once in 25 mM Tris buffer. Kinase reactions were carried out in 40 μl of kinase buffer (25 mM Tris-HCl, pH 7.5, 10 mM MgCl_2_, 1 mM dithiothreitol) containing 10 μCi of [γ-^32^P]ATP (3000 Ci mmol^−1^; PerkinElmer Life Sciences) and myelin basic protein (MBP), GST-E-cad-cytoplasmic domain, or GST-β-catenin at 25°C for 20 minutes. Reactions were terminated by the addition of SDS sample buffer, and the ^32^P-incorporated proteins were fractionated by SDS-polyacrylamide gel electrophoresis and visualized by autoradiography. The radioisotope activity was quantified using a phosphoimager system (Pharmacia).

### *In vitro* phosphorylation and dephosphorylation of E-cadherin cytoplasmic domain

To phosphorylate GST-E-cad-cytoplasmic domain (cd) by GFP-PKCδ, GFP-PKCδ transiently overexpressed in HEK293 cells was immunoprecipitated by anti-GFP and suspended in 40 μl of kinase buffer (25 mM Tris-HCl, pH 7.5, 10 mM MgCl_2_) containing 1 mM ATP and 0.5 μg purified GST-E-cad (cd) at 25°C for 20 minutes. To dephosphorylate GST-E-cad (cd) by calf intestine phosphatase (CIP), the supernatant containing phosphorylated GST-E-cad (cd) was transferred to a new tube and incubated with 2 μl (20 units) CIP in CIP buffer (100 mM Nacl, 50 mM Tris-HCl, pH 7.5, 10 mM MgCl_2_, 1 mM dithiothreitol) at 37°C for 60 minutes. The GST-E-cad (cd) was analyzed by immunoblotting with anti-E-cad pT790.

### Biotinylation of cell surface proteins

The cells were grown in a 100-mm dish to confluence and were washed 3 times with ice-cold PBS and incubated with 0.5 mg/ml sulfo-NHS-biotin in PBS at room temperature for 30 min. Cells were then washed 3 times with PBS and lysed in 1% Nonidet P-40 lysis buffer, and lysates were affinity-precipitated with 20 μl of avidin-immobilized agarose beads for 1 h at 4°C. After being washed three times in Nonidet P-40 lysis buffer, the beads were subjected to immunoblotting with anti-E-cadherin or anti-β-catenin.

### *In vitro* binding assay for purified β-catenin and E-cadherin

One hundred ng of purified β-catenin was incubated with 0.5 μg of purified GST or GST-E-cadherin-cytoplasmic domain (cd) in PBS at 4°C for 1.5 h. The protein complexes were pulled-down by glutathione agarose beads and washed three times in PBS with 1% Triton. The protein complexes were analyzed by immunoblotting with anti-β-catenin.

### Assay for homophilic interaction of E-cadherin

One μg of E-cadherin/Fc chimera protein (10 μg/ml) in phosphate buffered saline (PBS) containing 0.1 mM CaCl_2_ and 0.1 mM MgCl_2_ was added to each well of 96-well plates at 4°C for 24 h. Cells (5×10^5^) were suspended in serum free medium and plated onto the 96-well plates coated with the E-cadherin/Fc chimera protein at 37°C for 2 h. The 96-well plates were washed with PBS to remove non-adherent cells. The adherent cells were stained with 3-(4,5-cimethylthiazol-2-yl)-2,5-diphenyl tetrazolium bromide (MTT) and lysed in PBS containing 20% SDS. The value of absorbance at 595 nm was measured by the ELx800 absorbance microplate reader (Bio-Tek Instruments, Inc.).

### Assays for cell scattering and cell aggregation

For the cell scatter assay, MDCK cells were allowed to grow as colonies on 60-mm dish. When the colonies contained approximately 20 cells, the growth medium containing 10% serum was replaced with fresh medium containing 2% serum and 20 ng/ml HGF. After 12 h of HGF stimulation, the cell colonies were fixed and stained with Giemsa stain. Digital images of the colonies were taken under a microscope and the percentage of scattered colonies out of all counted colonies (n=100) was determined. A colony was judged as ‘scattered’ when the half of the cells in the colony had lost contact with their neighbors and exhibited a fibroblast-like phenotype. For the cell aggregation assay, cells were collected by trypsinization, suspended in DMEM supplemented with 10% serum at 10^6^ cells/ml and subjected to a constant rotation at 0.5 × g in a CO_2_ incubator. Two days later, the number of cell aggregates with a diameter of 400 μm or larger was measured under a phase contrast microscope at 40× magnification.

### Laser-scanning confocal fluorescent microscopy

For immunofluorescence staining, the cells were fixed for 15 min in PBS containing 4% paraformaldehyde, and permeabilized in PBS containing 0.5% Triton X-100 for 15 min. Coverslips were stained with primary antibodies at 4°C overnight, followed by TRITC-conjugated, Alexa Fluor 488-conjugated, Alexa Fluor 546-conjugated or Cy5-conjugated secondary antibodies (Jackson ImmunoReseach Laboratories) at 4 μg/ml for 120 min. The primary antibodies used for immunofluorescence staining were diluted before use: anti-E-cadherin (ECCD2, 1:200; clone 36, 1:200), anti-PKCδ (C-17, 1:100) and anti-Met (DL21, 1:200). Two μM TRITC-conjugated phalloidin was used to stain actin filaments. Coverslips were mounted in mounting medium (Anti-Fade Dapi-Fluoromount-G; SouthernBiotech). The images were acquired using a laser-scanning confocal microscope imaging system (LSM 510; Carl Zeiss) with a Plan Apochromat 63× (NA 1.2 W Korr; Carl Zeiss). Z sections (Figure 1A) were acquired at 0.5-μm steps. The images were cropped in Photoshop CS5 (Adobe) and were assembled by Illustrator CS2 (Adobe). The profiles of fluorescence intensity (Figure 5B) were depicted with line-graphs using LSM510 software (Carl Zeiss).

### 3D structure presentation

The structure of full-length murine β-catenin and cytoplasmic domain of murine E-cadherin complex (PDB ID code: 1I7X) was retrieved from RCSB Protein Data Bank (http://www.rcsb.org/pdb/home/home.do) and was visualized by Discovery Studio 3.1 software (Accelrys Inc., San Diego, USA). All bound water and non-protein atoms were removed from the complex. The Thr790 residue of E-cadherin was simulated in the phosphorylated state.

### Surgical specimens

We enrolled 7 cases with paired-frozen tissues of cervical carcinoma and adjacent noncancer epithelia for immunoblotting analysis. All these cases are patients with early-stage (International Federation of Gynecology and Obstetrics staging Ib) cervical cancer who underwent radical hysterectomy and pelvic lymphadenectomy at National Cheng Kung University Hospital, Taiwan. The collection of surgical specimens was approved by the institutional review board of National Cheng Kung University Hospital.

### Statistics

Statistical analyses were performed using Student's *t* test. Differences were considered to be statistically significant at *P* < 0.05.

## SUPPLEMENTARY INFORMATION FIGURES



## Abbreviations

PKC: protein kinase C
GFP: green fluorescent protein
MDCK: Madin-Darby canine kidney
HGF: hepatocyte growth factor
shRNA: short-hairpin RNA
CHO: Chinese hamster ovary
GST: glutathione-S- transferase
MBP: myelin basic protein
