# Supplementary material for: Phosphorylation of E-cadherin at threonine 790 by protein kinase Cδ reduces β-catenin binding and suppresses the function of E-cadherin
Source: Oncotarget. 2016 May 17;7(24):37260–76. doi: 10.18632/oncotarget.9403 (PMC5095074; doi:10.18632/oncotarget.9403)
Supplement: Supplementary file 1 [file oncotarget-07-37260-s001.pdf]

Phosphorylation of E-cadherin at threonine 790 by protein kinase Cδ reduces β-catenin binding and suppresses the function of E-cadherin

SUPPLEMENTARY INFORMATION

Online supplemental information

Figure S1 shows that Gö6976, a selective inhibitor for the classical PKC isozymes, does not inhibit the phosphorylation of E-cadherin Thr790 and the scatter of MDCK cells upon HGF stimulation.
Figure S2 and S3 show larger fields for Figure 5C and Figure 6F, respectively.

SUPPLEMENTARY FIGURES

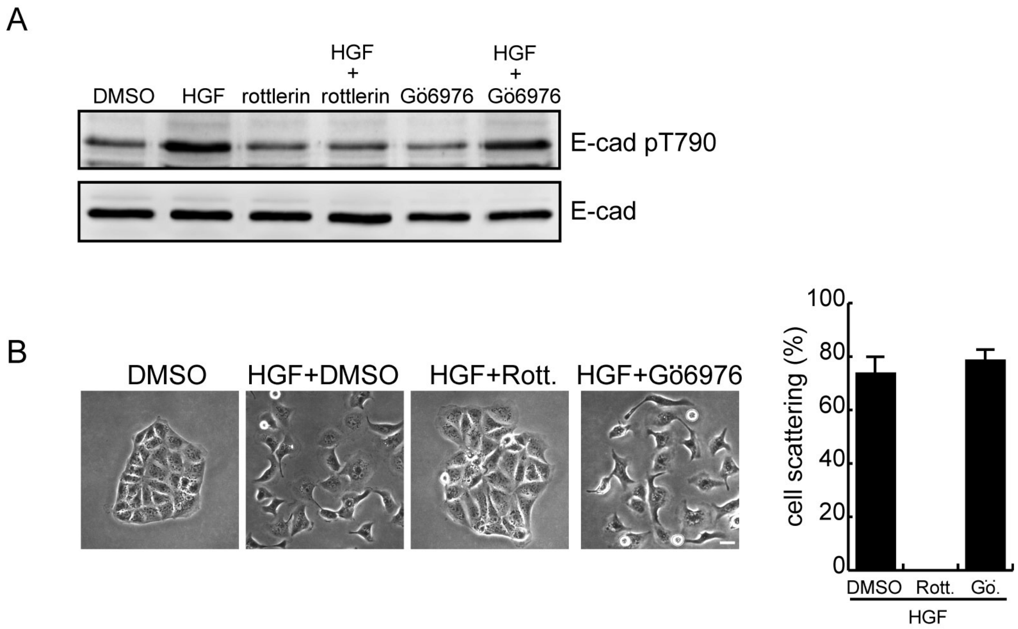

Supplementary Figure S1: Classical PKCs are likely not involved in the phosphorylation of E-cadherin T790 and the cell scattering induced by HGF. A. MDCK cells were serum-starved for 24 h and were then treated with HGF (20 ng/ml) for 15 min in the presence of rottlerin (5 μM) or Gö6976 (1 μM). The cell lysates were analyzed by immunoblotting with antibodies to E-cadherin and E-cadherin pT790. **B.** MDCK cells were allowed to grow as colonies and were then treated with HGF (20 ng/ml) in the presence of rottlerin (5 μM) or Gö6976 (1 μM). The solvent dimethy sulphoxide (DMSO) was used as a control. Twelve hours later, the percentage of scattered colonies out of the total counted cell colonies (n ≥ 100) was determined. The values (mean ± SD) are from three experiments. The representative micrographs were taken under a phase-contrast microscope. The scale bar represents 20 μm.

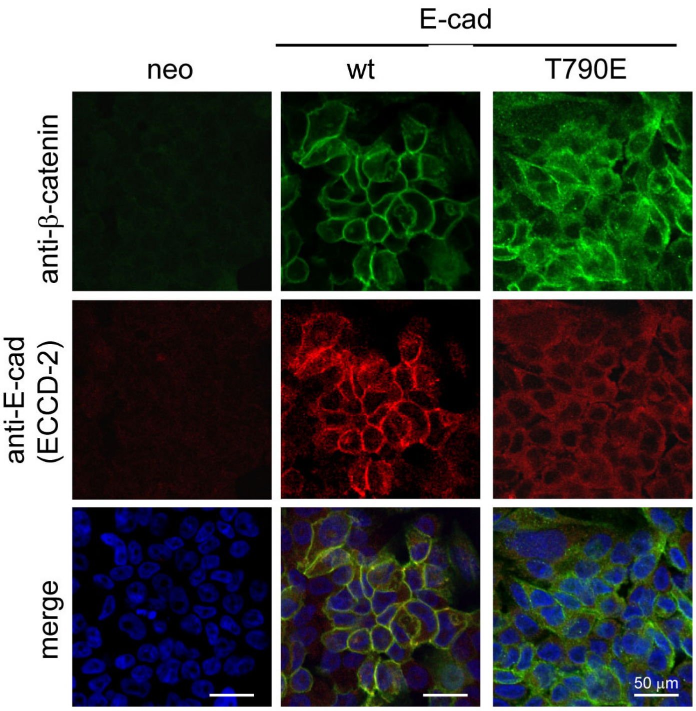

Supplementary Figure S2: β-catenin is less organized at the cell-cell junctions of the CHO cells expressing the E-cadherin T790E mutant compared to those expressing the wt E-cadherin. CHO cells stably expressing E-cadherin wt or T790E were grown to confluence and were then stained with anti-β-catenin and anti-E-cadherin (ECCD-2). Note that β-catenin is less organized at the cell-cell contacts of the CHO cells expressing E-cadherin T790E. The scale bar represents 50 μm.

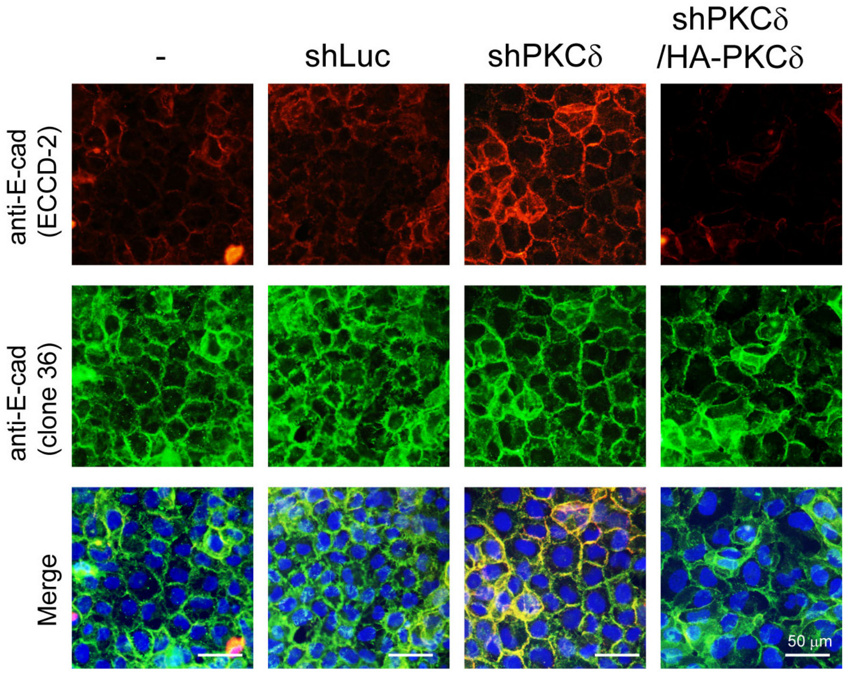

Supplementary Figure S3: The depletion of PKCδ in the CaSki clone #1 cells enhances the homophilic interactions of E-cadherinCaSki #1 cells were infected with lentiviruses expressing shRNAs to PKCδ (shPKCδ) or luciferase (shLuc) as a control. HA epitope tagged-PKCδ (HA-PKCδ) was re-expressed in the cells whose endogenous PKCδ had been depleted. The cells were grown to confluence and were then stained with anti-E-cadherin antibodies (clone 36 and ECCD-2). The scale bar represents 50 μm.
